# Efficacy of *Lactococcus lactis* 11/19-B1 in a Mouse Model of Allergic Rhinitis

**DOI:** 10.1055/a-2804-6274

**Published:** 2026-08-14

**Authors:** Toshihiko Suzuki, Nozomu Miyazaki, Chiaki Ozaki, Mitsuhiro Okano, Hiroshi Ogawa, Tatsuo Suzutani

**Affiliations:** 1 Department of Otolaryngology, Fukushima Medical University Aizu Medical Center, Fukushima, Japan; 2 Department of Microbiology, Fukushima Medical University, Fukushima, Japan; 3 Department of Otorhinolaryngology, International University of Health and Welfare School of Medicine, Narita, Japan; 4 Ohara General Hospital, Fukushima, Japan

**Keywords:** Lactococcus lactis, allergic rhinitis, animal model, eosinophils, helper T cells

## Abstract

**Introduction:**

Lactococcus lactis (L. lactis) is widely used in fermented foods and has immunomodulatory properties. However, its effects on allergic rhinitis remain unclear.

**Objective:**

This study evaluated the efficacy of L. lactis 11/19-B1 in mitigating allergic rhinitis symptoms in a mouse model.

**Methods:**

Female BALB/c mice were fed a diet supplemented with heat-killed L. lactis 11/19-B1, while allergic rhinitis was induced via intraperitoneal and intranasal administration of ovalbumin (OVA). The positive control group received a standard diet without L. lactis 11/19-B1. Sneezing frequency was recorded, and tissue samples were collected for analysis. Serum OVA-immunoglobulin E (IgE) levels were also measured, nasal mucosa histopathology assessed, and the interleukin 4 (IL-4), interferon gamma (IFN-γ), T helper (Th) 1/Th2 balance in cluster of differentiation (CD) 4+ T cells analyzed by flow cytometry (FCM).

**Results:**

Mice receiving L. lactis 11/19-B1 showed significantly reduced sneezing frequency and decreased eosinophil infiltration in the nasal mucosa. No significant changes in serum OVA-IgE levels were observed. Flow cytometry analysis showed a significant increase in the Th1/Th2 ratio in Peyer's patches in the L. lactis 11/19-B1 group compared with the positive control group.

**Conclusion:**

These findings suggest that L. lactis 11/19-B1 alleviates allergic rhinitis symptoms by modulating the Th1/Th2 balance and reducing eosinophil infiltration, independently of IgE suppression. These findings highlight its potential as a dietary supplement for allergic disease management .

## Introduction

Allergic rhinitis is a significant public health concern, affecting more than 400 million individuals globally. Prevalence rates range from 10% to 30% among adults and as high as 40% in children. 1 In Japan, the prevalence of allergic rhinitis has been steadily rising, reaching 49.2% in 2019. 2 This condition, characterized by symptoms such as nasal congestion, sneezing, and itching, poses a substantial burden on both patients' quality of life and healthcare systems.

The primary treatments for allergic rhinitis include antihistamines, glucocorticosteroids, mast cell stabilizers, decongestants, and leukotriene receptor antagonists. 3 4 5 6 While these therapies are effective in symptom management, their use is often accompanied by side effects. For instance, H1-antihistamines can cause cardiotoxicity, central nervous system depression, and anticholinergic effects. 7 Long-term glucocorticosteroid use carries risks such as skin disorders, menstrual irregularities, and adrenal insufficiency. 8 Leukotriene receptor antagonists may result in dermatological complications, including rashes and vasculitic lesions. 9 These limitations underscore the need for safer, more tolerable treatment alternatives.

Recent attention has turned to the potential of lactic acid bacteria (LAB) in managing allergic rhinitis. Several LAB strains have demonstrated promising effects. For example, *Lactobacillus acidophilus* ( *L. acidophilus* ) L-55 has been shown to alleviate symptoms in patients with cedar pollen allergies, reduce sneezing and nasal rubbing in murine models, and suppress serum immunoglobulin E (IgE) production. 10 11 Similarly, *L. acidophilus* L-92 has been reported to relieve perennial allergic rhinitis symptoms. 12 LABs are generally considered safe, 13 and even their inactivated forms retain some functional properties. 14 Their anti-allergic potential has already been harnessed in various functional foods, but the specific effects and mechanisms of individual strains remain incompletely understood.

Most research on probiotic LABs has focused on *Lactobacillus* and *Bifidobacterium* , which are commonly isolated from the human or animal intestinal tract. 15 However, Lactococcus lactis (L. lactis), a lactic acid bacterium widely used in fermented foods such as yogurt and cheese, has also been shown to regulate the host immune system. 16 Among L. lactis strains, L. lactis 11/19-B1, isolated from the surface of kiwifruit, has demonstrated unique immunomodulatory properties. 17 Studies have demonstrated that this strain enhances innate immunity in silkworms, increasing resistance to *Pseudomonas aeruginosa* infections. 17 In humans, the consumption of yogurt containing *L. lactis* 11/19-B1 has been associated with reductions in low-density lipoprotein (LDL) levels and activation of cellular immunity. 18 Notably, this strain is capable of surviving passage through the intestines, and its sensitivity to various antibiotics minimizes the risk of transferring drug resistance to other gut bacteria. Furthermore, as *L. lactis* 11/19-B1 does not produce bacteriocins, it is unlikely to disrupt the balance of the beneficial intestinal microbiota, thereby reducing the risk of dysbiosis. 19

Importantly, in atopic dermatitis patients, the ingestion of *L. lactis* 11/19-B1 led to improvements in skin symptoms, with histopathological studies showing inhibited eosinophil infiltration in animal models. 20 Eosinophil infiltration, a hallmark of late-phase allergic reactions, 21 plays a critical role in histamine release and symptom exacerbation in allergic rhinitis. 22 Targeting eosinophil activity offers a promising approach to symptom alleviation.

Despite its demonstrated immunomodulatory effects, the impact of *L. lactis* 11/19-B1 on allergic rhinitis has not yet been explored. In this study, we investigated whether *L. lactis* 11/19-B1 can alleviate allergic rhinitis symptoms and modulate the immune response in a mouse model, providing new insights into its potential for the safe and effective clinical management of allergy symptoms.

## Methods

### Animals

The experimental protocol complied with the ARRIVE (Animal Research: Reporting In Vivo Experiments) guidelines and was reviewed and approved by the Fukushima Medical University Animal Ethics Committee (permission No. 2021004). Twenty-four three-week-old female Bagg's Albino/c (BALB/c) mice (10–15 g) were randomly assigned to three groups (n = 8 per group): the *L. lactis* 11/19-B1 group, the positive control group, and the negative control group. Mice were housed under standard laboratory conditions with ad libitum access to water and food.

### Lactic Acid Bacteria Treatment

*L. lactis* 11/19-B1 was cultured at 37°C for 24 hours, washed twice with 0.85% NaCl, suspended in sterilized distilled water, heat-killed at 100°C for 30 minutes, and lyophilized. The *L. lactis* 11/19-B1 group was fed a CE-2 diet containing 0.05% *L. lactis* 11/19-B1, while the positive and negative control groups received a standard CE-2 diet without *L. lactis* 11/19-B1. The *L. lactis* 11/19-B1 preparation contained 5 × 10 ^8^ CFU/mg.

### Ovalbumin (OVA) Sensitization and Challenge

**Fig. 1:**
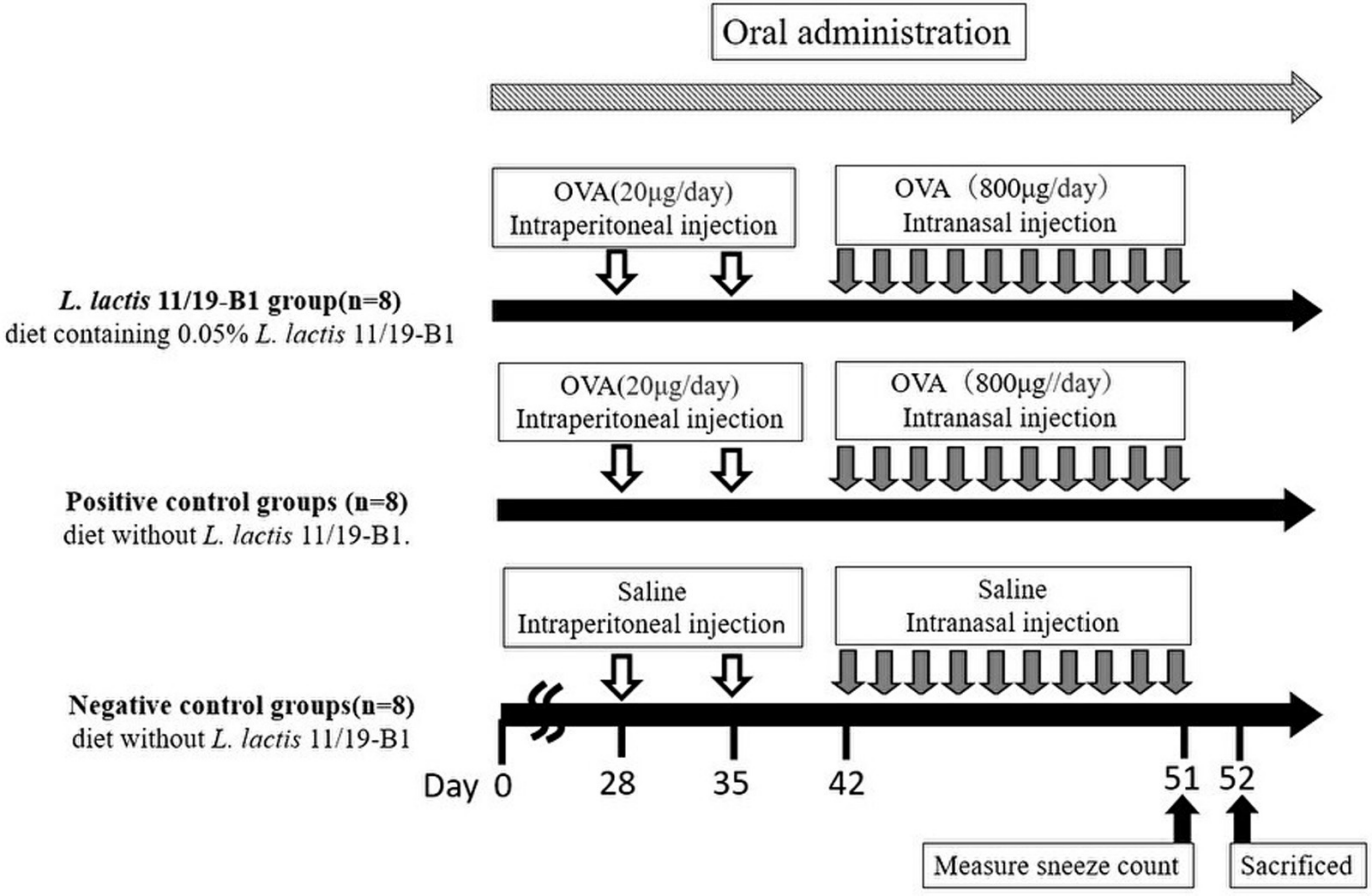
The experimental schedule. L. lactis 11/19-B1, Lactococcus lactis 11/19-B1; OVA, ovalbumin.

Mice in the *L. lactis* 11/19-B1 and positive control groups were sensitized on Days 28 and 35 with intraperitoneal injections of 20 μg OVA in 0.1 mL of 2 mg Al(OH) _3_ gel. From days 42 to 51, mice received daily intranasal challenges with 40 mL of OVA solution (20 mg/mL). In the negative control group, saline was administered intraperitoneally and intranasally. On day 53, all mice were euthanized, and tissue and blood samples were collected for analysis. A schematic overview of the study design is presented in Fig. 1 .

### Observation of Nasal Symptoms

To evaluate nasal symptoms, the number of sneezes was recorded by video for 10 minutes following the final intranasal challenge on Day 52. Videos were reviewed by a blinded observer to count the total number of sneezes.

### Measurement of Serum OVA-Specific IgE Levels

Serum OVA-specific IgE levels were quantified using a Mouse IgE ELISA kit (Cayman Chemical, Ann Arbor, MI, USA) according to the manufacturer's protocol.

### Flow Cytometry

Cells were isolated from the spleen, mesenteric lymph nodes (MLNs), and Peyer's patches (PPs). For surface marker analysis, cells were washed with ice-cold flow cytometry (FCM) buffer (1% BSA in PBS) and incubated with CD3-PE (145-2C11, eBioscience, CA, USA), CD4-FITC (RM4-5, BioLegend, SD, USA), and CD25-PE (PC61.5, eBioscience) antibodies for 30 minutes. After washing twice, data were obtained using a FACSCanto II flow cytometer (BD Biosciences) and analyzed with FlowJo software (Tree Star Inc., Ashland, OR, USA).

For intracellular cytokine staining, cells were stimulated at 37°C for 4 hours (interleukin 4, IL-4) or 16 hours (interferon gamma, IFN-γ) using a Cell Stimulation Cocktail with protein transport inhibitors (eBioscience). Fixation and permeabilization were performed with IC Fixation and Permeabilization Buffers (Invitrogen). Staining was completed with IL-4-APC (11B11, eBioscience) or IFN-γ-APC (XMG1.2, BioLegend) antibodies. Forkhead box P3 (FOXP3) staining was conducted using the FOXP3 Fix/Perm Buffer Set (BioLegend) and FOXP3-APC (FJK-16s, eBioscience) according to the manufacturer's instructions.

### Histological Evaluation

Nasal tissues were collected for histological analysis. Mouse heads were dissected, and the skin was removed. Skulls were decalcified in 10% ethylenediaminetetraacetic acid (EDTA) for 10 days, post-fixed in 10% formalin for 2 days, and embedded in paraffin. Serial sections (5 μm) were prepared at the level of the nasal septum's trailing end and stained with direct fast scarlet (DFS) to detect eosinophils. 23 Sections were examined using light microscopy at 400× magnification.

### Statistical Analysis

Statistical analyses were performed using GraphPad Prism version 6.07 (GraphPad Software Inc., San Diego, CA, USA). Results are expressed as the mean ± standard error of the mean (SEM). One-way analysis of variance (ANOVA) followed by Tukey's multiple comparison test was used for group comparisons. A p-value of < 0.05 was considered statistically significant.

## Results

### Sneeze Frequency

**Fig. 2:**
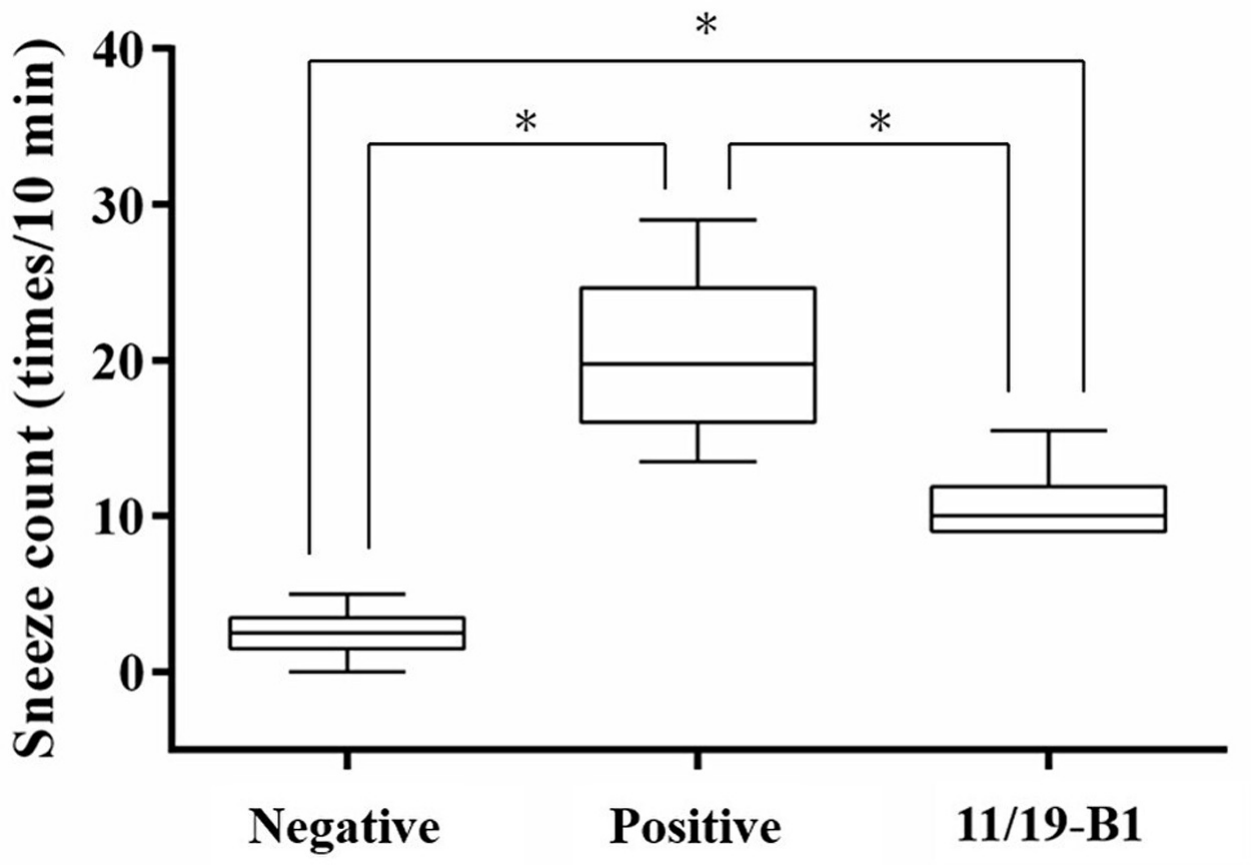
Number of sneezes following the final intranasal challenge on day 52 in the L. lactis 11/19-B1, positive control, and negative control groups. Negative, negative control group; Positive, positive control group; 11/19-B1, L. lactis 11/19-B1 group. *p < 0.05.

The effect of *L. lactis* 11/19-B1 on sneeze frequency induced by intranasal OVA challenge is shown in Fig. 2 . Oral administration of L. lactis 11/19-B1 significantly reduced the number of sneezes compared with the positive control group (p < 0.05) . The negative control group exhibited a substantially lower sneeze frequency than both the positive control and *L. lactis* 11/19-B1 groups (p < 0.05).

### Serum OVA-Specific IgE

**Fig. 3:**
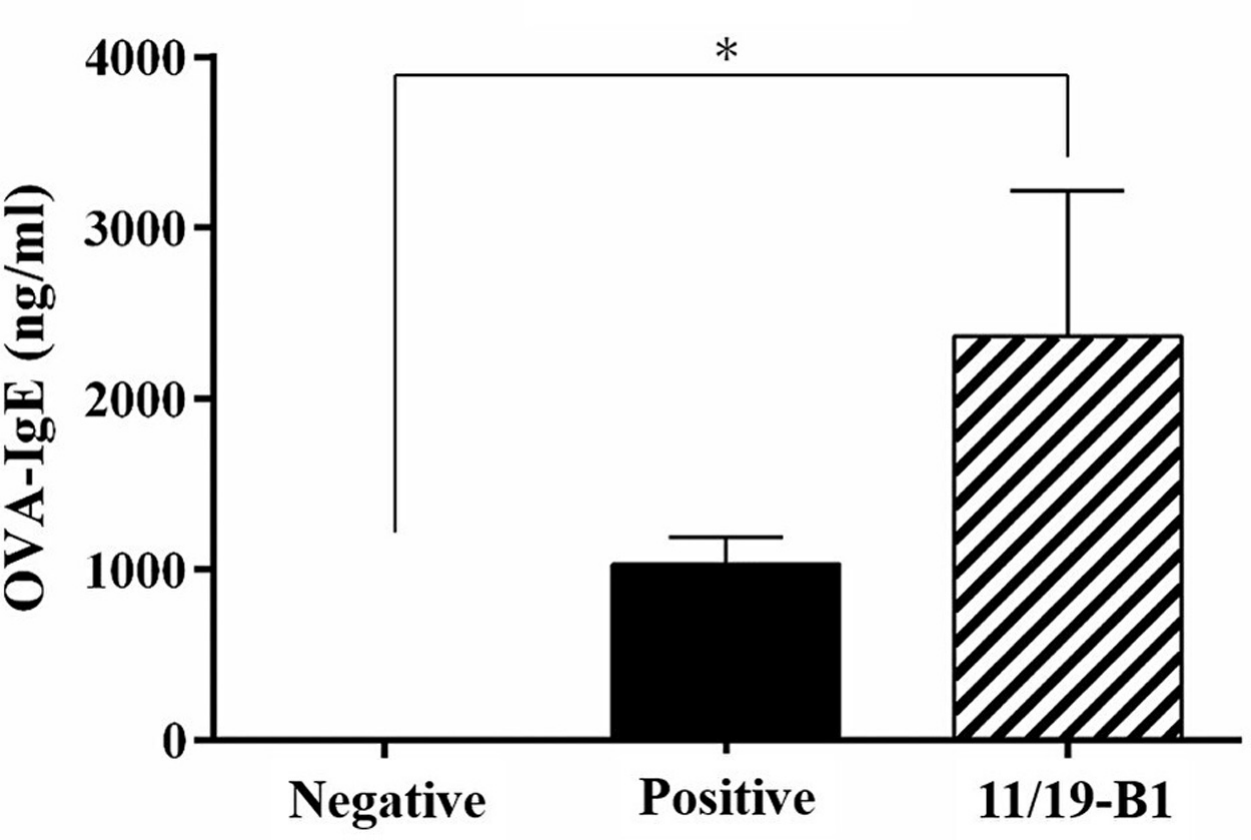
Serum OVA-specific IgE levels in the L. lactis 11/19-B1 group, the positive control group, and the negative control group. Negative, negative control group; Positive, positive control group; 11/19-B1, Lactococcus lactis 11/19-B1group. *p < 0.05.

The levels of OVA-specific IgE are presented in Fig. 3 . Both the *L. lactis* 11/19-B1 group and the positive control group showed significantly elevated OVA-specific IgE levels compared with the negative control group (p < 0.05). However, there was no significant difference in IgE levels between the *L. lactis* 11/19-B1 and positive control groups.

### Histological Changes

**Fig. 4:**
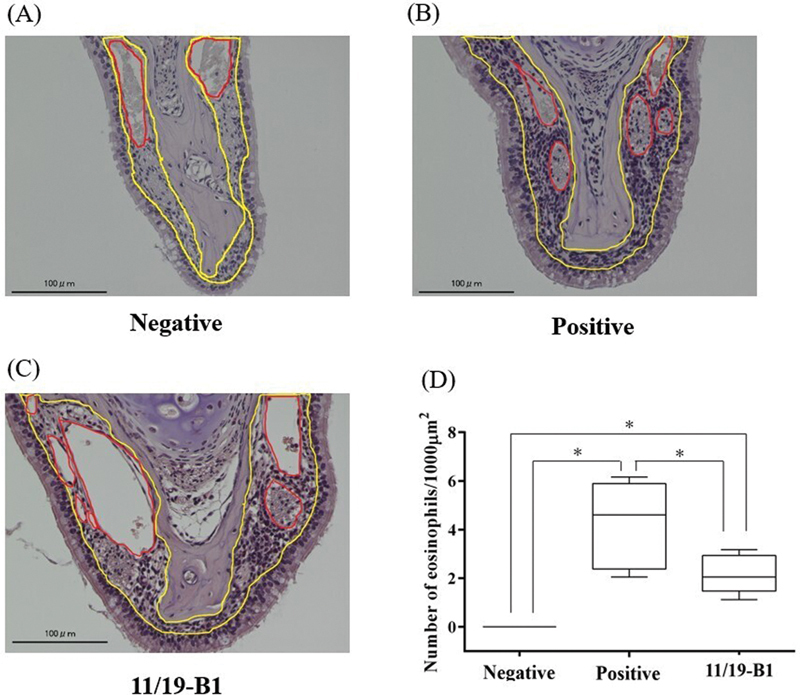
Posterior nasal septum stained with DFS. The area surrounded by the yellow line is the submucosal tissue. The area surrounded by the red line is the blood vessels. ( **A** ) The negative control group. ( **B** ) The positive control group. ( **C** ) L. lactis 11/19-B1 group. ( **D** ) Comparison of the density of eosinophils per area in the submucosa excluding vascular areas. Negative, negative control group; Positive, positive control group; 11/19-B1, L. lactis 11/19-B1 group. *p < 0.05.

Eosinophil density in the submucosa of the posterior nasal septum, excluding vascular areas, was evaluated using DFS staining ( Fig. 4 ). As shown in Fig. 4(D) , the eosinophil density in the negative control group was significantly lower than that in both the positive control and *L. lactis* 11/19-B1 groups (p < 0.05). Additionally, the *L. lactis* 11/19-B1 group exhibited a significantly lower eosinophil density compared with the positive control group (p < 0.05).

### Flow Cytometry

**Fig. 5:**
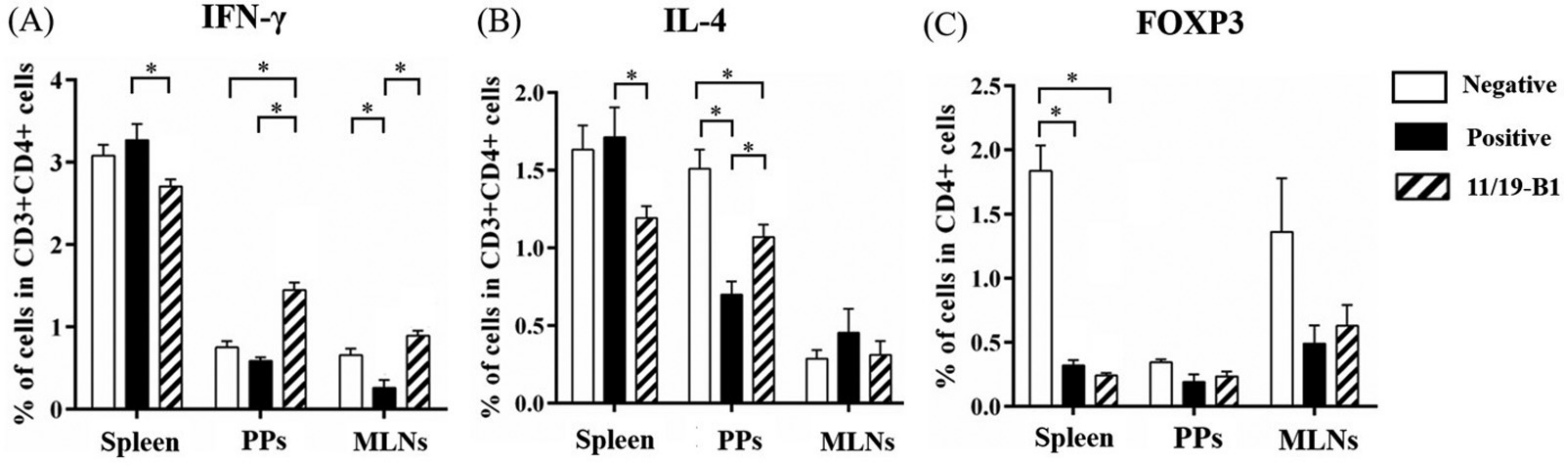
Percentages of IL-4+ (A), IFN-g+ (B), and FOXP3+ (C) CD4+ T cells in the spleen, Peyer's patches, and mesenteric lymph nodes in each group. PPs, Peyer's patches; MLNs, mesenteric lymph nodes; Negative, negative control; Positive, positive control; 11/19-B1, L. lactis 11/19-B1 group. *p < 0.05.

The levels of IFN-γ and IL-4 in the spleen, PPs, and MLNs were analyzed ( Fig. 5 ). In the *L. lactis* 11/19-B1 group, IFN-γ levels were significantly increased in the PPs and MLNs compared with the positive control group (p < 0.05) but were significantly decreased in the spleen (p < 0.05). IL-4 levels were significantly decreased in the spleen (p < 0.05) and non-significantly reduced in the MLNs, while showing a significant increase in the PPs (p < 0.05). There was no significant difference in FOXP3 expression between the *L. lactis* 11/19-B1 and positive control groups.

### T helper (Th) 1/Th2 Ratio

**Fig. 6:**
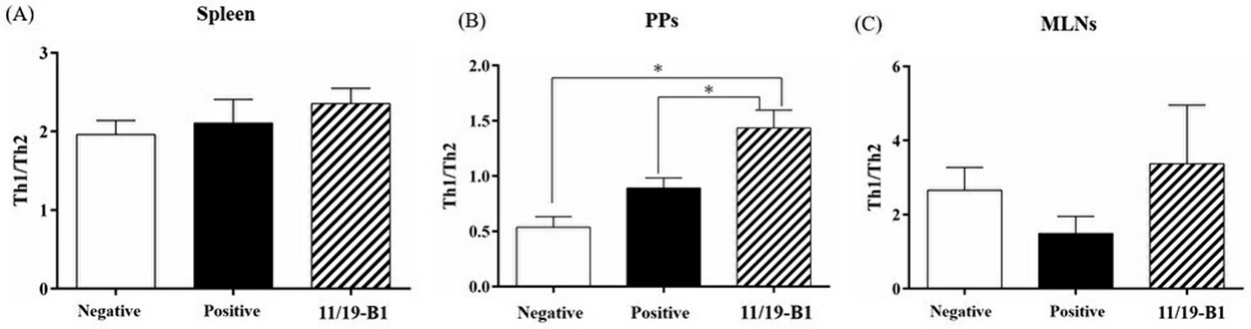
Th1/Th2 ratio in the spleen (A), PPs (B), and MLNs (C) of mice in each group. PPs, Peyer's patches; MLNs, mesenteric lymph nodes; Negative, negative control; Positive, positive control; 11/19-B1, L. lactis 11/19-B1 group. *p < 0.05.

The Th1/Th2 ratios in the spleen, PPs, and MLNs are shown in Fig. 6. The *L. lactis* 11/19-B1 group displayed a significantly higher Th1/Th2 ratio in the PPs compared with the positive control group (p < 0.05). Although the Th1/Th2 ratios in the spleen and MLNs were also higher in the *L. lactis* 11/19-B1 group, the differences were not statistically significant.

## Discussion

This study demonstrated that *L. lactis* 11/19-B1 effectively reduced sneezing frequency in a mouse model of allergic rhinitis. Importantly, this reduction occurred without a decrease in IgE levels, suggesting the involvement of mechanisms independent of IgE suppression. Our previous research also showed that this strain alleviated symptoms of atopic dermatitis in a mouse model without suppressing IgE production, 20 suggesting that L. lactis 11/19-B1 may exert its effects through IgE-independent pathways. In the present study, the observed reduction in eosinophil infiltration into the nasal mucosa is likely a key factor contributing to the suppression of sneezing.

Eosinophils play a critical role in histamine release. Mediators derived from eosinophils, such as major basic protein (MBP) and eosinophil cationic protein (ECP), can stimulate mast cells to release histamine. 21 In this study, the decrease in eosinophil infiltration into the nasal mucosa may have reduced histamine release, suppressing sneezing. As eosinophil infiltration into the nasal mucosa is a hallmark of late-phase allergic reactions, *L. lactis* 11/19-B1 may act to modulate this process.

Allergic rhinitis is characterized by an excessive Th2 response. The Th2 cytokines IL-4, IL-5, and IL-13 play important roles in eosinophil migration. 24 25 The present study found that *L. lactis* 11/19-B1 reduced the proportion of IL-4-producing T cells in the mesenteric lymph nodes and spleen, suppression of the Th2 response. Additionally, the observed changes in PPs highlight the potential role of gut-associated lymphoid tissue in systemic immune modulation. This is consistent with the hypothesis that oral administration of *Lactococcus* influences the immune system through the gut, subsequently affecting systemic immunity. 15 26 27

Moreover, *L. lactis* 11/19-B1 promoted a shift in the Th1/Th2 balance toward a Th1-dominant response, which likely contributed to the suppression of Th2-driven eosinophil recruitment and reduced local allergic reactions. Unlike other LAB strains such as L. acidophilus L-55 and L-92, which primarily act via IgE suppression, L. lactis 11/19-B1 appears to exert its anti-allergic effects through IgE-independent pathways . This highlights its unique mechanism and potential advantage for specific allergic conditions where IgE suppression is less effective.

However, the reduction of IFN-γ levels in the spleen in the *L. lactis* 11/19-B1 group remains unclear and warrants further investigation. In addition to its effects on the Th1/Th2 balance and eosinophils, other immune cells such as dendritic cells (DCs) and regulatory T cells (Tregs) are known to play crucial roles in allergic diseases. 28 29

While no significant changes in Treg were observed in this study, the potential influence of *L. lactis* 11/19-B1 on DC activation and function warrants further investigation. Understanding its broader impact on immune cell networks could provide a more comprehensive view of its mechanisms in allergy suppression.

The present study employed heat-killed bacteria; future studies using live bacteria could provide further insights into the allergy-suppressing mechanisms of L. lactis 11/19-B1. Future studies should focus on directly measuring histamine levels and evaluating the effects of L. lactis 11/19-B1 on the high-affinity IgE receptor (FceRI) on mast cell surfaces, as well as histamine H1 and H4 receptor expression, to clarify the pathways involved. 30 31 32

## Conclusion

*L. lactis* 11/19-B1 appears to modulate the Th1/Th2 balance, suppress eosinophil infiltration, and alleviate allergic rhinitis symptoms. These findings highlight the potential of *L. lactis* 11/19-B1 as a functional food ingredient for managing allergic rhinitis. Building on these findings, future studies evaluating the efficacy and safety of L. lactis 11/19-B1 in human clinical trials will be important . Such studies could provide valuable data on the potential of L. lactis 11/19-B1 as a dietary intervention for allergic rhinitis and other allergy-related conditions. Additionally, the long-term effects of its consumption, its potential for preventing other allergic responses, and its comparison with existing probiotics should be explored to establish its utility in clinical and dietary settings.
